# Sol-Gel-Prepared Ni-Mo-Mg-O System for Catalytic Transformation of Chlorinated Organic Wastes into Nanostructured Carbon

**DOI:** 10.3390/ma13194404

**Published:** 2020-10-02

**Authors:** Grigory B. Veselov, Timofey M. Karnaukhov, Yury I. Bauman, Ilya V. Mishakov, Aleksey A. Vedyagin

**Affiliations:** 1 Department of Materials Science and Functional Materials, Boreskov Institute of Catalysis SB RAS, 630090 Novosibirsk, Russia; andreyveselo@yandex.ru (G.B.V.); karnaukhovtm@catalysis.ru (T.M.K.); bauman@catalysis.ru (Y.I.B.); mishakov@catalysis.ru (I.V.M.); 2 Department of Natural Sciences, Novosibirsk State University, 630090 Novosibirsk, Russia

**Keywords:** ternary oxide system, catalytic activity, CCVD, particles’ distribution, filamentous carbon formation

## Abstract

The present work aimed to prepare Ni-Mo particles distributed within the MgO matrix. With this purpose in mind, a ternary Ni-Mo-Mg oxide system was synthesized by a sol-gel approach. The samples were studied by low-temperature nitrogen adsorption, X-ray diffraction analysis, and transmission electron microscopy equipped with energy dispersive X-ray analysis. Both the nickel and molybdenum species in the prepared samples were characterized by a fine and uniform distribution. The diffraction pattern of the ternary system was predominantly represented by the MgO reflections. The catalytic activity of the samples was tested in the decomposition of 1,2-dichloroethane used as a representative of the chlorinated organic wastes. The nanostructured carbon filaments resulting from the decomposition of the halogenated substrate were found to be characterized by a narrow diameter distribution, according to the transmission electron microscopy data, thus confirming the fine distribution of the active Ni-Mo particles. The results obviously show the advantages of the sol-gel technique for obtaining efficient catalysts.

## 1. Introduction

Nowadays, nickel-molybdenum oxide systems are widely applied in a variety of technological processes. Being supported on oxides with a developed porous structure, they play a major role in hydrodenitrogenation of different nitrogen-containing organic compounds [1,2] and hydrodesulfurization of thiophenes [3,4]. In addition to the mentioned processes, the nickel-molybdenum catalysts exhibit the activity in steam reforming and oxidative conversion of hydrocarbons [5,6,7,8]. As reported, the addition of small amounts of molybdenum oxide noticeably decreases the rate of the coke formation over the catalyst’s surface, thus increasing its activity [5,8]. Wang et al. [9] studied the process of oxidative dehydrogenation of propane over Ni-Mo-Mg-O catalysts and found that the Ni:Mo ratio is a crucial parameter and provides the best performance at 1:1. On the other hand, the Ni-Mo oxide systems are attractive as catalysts for the preparation of various carbon materials, including carbon nanotubes [10,11,12,13,14,15,16,17] and nanofibers [18,19], via catalytic chemical vapor deposition or combustion. In this case, the addition of molybdenum contrary increased the yield of carbon products and their specific surface area compared with pure nickel catalysts [20,21].

It is well known that in most fields of catalysis, the dispersion of the active components is the most important factor affecting the overall efficiency of the catalysts. Usually, such active components are represented by dispersed nanoparticles acting as catalytic sites [22]. In order to obtain the nickel-molybdenum oxide systems with appropriate dispersion and catalytic activity, different preparation approaches can be used. Thus, very often, impregnation of the support is characterized by a developed porous structure (for instance, γ-Al_2_O_3_) with solutions of nickel and molybdenum nitrates followed by the drying and calcination procedures [3,8]. Other techniques include co-precipitation of hydroxides from the solution of aluminum and nickel nitrates in the presence of molybdenum ions [7,19], thermal decomposition of the salts’ mixture [14,16], and the Pechini method [9,13]. In the latter case, citric acid is added into the joint solution of nickel and magnesium nitrates and ammonium heptamolybdate. The resultant is mixed and heated up to 60–70 °C for 6–12 h. Then the formed gel is dried and calcined.

The sol-gel technique provides wide possibilities for the synthesis of multicomponent oxide systems with desired chemical properties. Thus, Gutić et al. [23] used the sol-gel method to obtain the Ni-Mo oxide precursor of Ni-Mo alloy powders for hydrogen evolution reaction. Umapathy et al. [24] prepared NiMoO_4_ nanoparticles for photocatalytic degradation purposes. An additional advantage of the sol-gel approach is the possibility to distribute uniformly active components within an oxide matrix or support. Baston et al. [25,26] incorporated the precursors of Mo, Co, and Ni oxides into the sol of γ-Al_2_O_3_ and ZrO_2_ being formed and found the developed surface area of the resulting materials, fine distribution of active components, and enhanced activity. Other research groups utilized the similar one-pot sol-gel route to synthesize Ni, Mo, Co, and Fe loaded MgO nanocatalysts [17,27,28]. In general, the obtained materials possess developed a porous structure and, as a result, high values of specific surface area (SSA) and pore volume (V_p_). In the case of the sol-gel derived from ternary Ni-Mo-Mg-O systems with an atomic Ni:Mo ratio of 1:1, the SSA and V_p_ reach values are 100 m^2^/g and 0.4 cm^3^/g, respectively [9,13]. The preparation method is technologically simple, and most of the stages can be managed in one reaction vessel. The products can be easily shaped into granules of different sizes and configurations.

Recently, we reported the application of the sol-gel method for the preparation of two-component MgO-based systems [29,30,31,32,33]. The preparation procedures included the hydrolysis of magnesium methoxide in a methanol/toluene solution, the drying of the formed gel in air (or supercritical drying), and the calcination of the xerogel in a muffle. Initially, the approach considered the hydrolysis of magnesium methoxide by water with the subsequent addition of an organic precursor of the second component [29]. In recent works, an aqueous solution of an inorganic precursor of the second component was used instead of pure water during the hydrolysis stage [30,31,32,33]. In the present research, this modified approach was applied to prepare the nanoparticles of Ni and Mo oxides distributed within the MgO matrix. The prepared samples were used as a catalyst for catalytic chemical vapor deposition (CCVD) of 1,2-dichloroethane with the formation of nanostructured carbon fibers. The choice of Ni and Mo as active components was not accidental. The Ni-based catalysts and bulk alloys were shown to be efficient for the utilization of the chlorinated organic wastes via their processing into segmented carbon nanofibers [34,35,36]. The addition of a textural promoter helped stabilize the nanoparticles in the dispersed state and control the size of the carbon nanofibers being formed [37]. Quite recently, it was found that the modification of Ni with Mo improves the catalytic performance of the catalyst and affects the structure and morphology of the formed carbon product [20,21]. Therefore, Ni and Mo oxide nanoparticles uniformly distributed within the MgO matrix, playing the role of textural promoter, were successfully prepared via the sol-gel technique, characterized by a set of physicochemical methods and tested in CCVD of 1,2-dichloroethane for the first time.

## 2. Materials and Methods

### 2.1. Synthesis of the Catalyst’s Samples

The specimen of the magnesium ribbon (purity of 99.9%, Sigma-Aldrich, St. Louis, MO, USA) was dissolved in methanol (Avantor Performance Materials, Gliwice, Poland) until formation of the saturated solution of magnesium methoxide. Toluene (Component-Reaktiv, Moscow, Russia) was used as a gel stabilizer and was added to the solution with a methanol:toluene ratio of 1:1. The hydrolysis was performed by a dropwise adding of an aqueous solution of salt precursors (Ni(NO_3_)_3_ (Baltic Enterprise, Saint-Petersburg, Russia) and (NH_4_)_2_MoO_4_ (Baltic Enterprise, Saint-Petersburg, Russia)) at intense stirring. The Ni:Mo ratio was 1:1. The total metal loading was 15 wt%, with respect to NiO and MoO_3_. The obtained gel was subjected to drying at room temperature for 2 h and then at 200 °C for another 2 h. Thus, prepared xerogel was denoted as Ni-Mo-Mg-OH. In order to obtain the oxide sample (labeled as Ni-Mo-Mg-O), the xerogel was calcined in air at smooth heating up to 500 °C.

### 2.2. Characterization of the Samples

The specific surface area of the samples was measured with the Brunauer-Emmett-Teller (BET) method. The low-temperature nitrogen adsorption was used in this case. The values of average pore diameter (D_av_) and pore size (D_pore_) distributions were determined with the Barrett-Joyner-Halenda (BJH) method from the nitrogen adsorption isotherms at 77 K by Digisorb-2600 (Quantochrome, Boynton Beach, FL, USA) and ASAP-2400 (Micrometrics, Norcross, GA, USA) instruments.

Scanning electron microscopic studies were performed using a JSM-6460 microscope (JEOL Ltd., Tokyo, Japan) with magnifications ranging from ×8 to ×300,000. A JEOL JEM-2010 electron microscope was used to obtain the transmission electron microscopy (TEM) images. The microscope possesses the lattice resolution of 0.14 nm and operates at an accelerating voltage of 200 kV. An EDAX spectrometer (Bruker Nano GmbH, Berlin, Germany) with an energy resolution of 128 eV was used to carry out the local energy-dispersion X-ray (EDX) microanalysis.

The X-ray diffraction (XRD) analysis was performed using an X’TRA (Thermo ARL) diffractometer (Thermo Fisher Scientific, Basel, Switzerland) (Bregg−Brentano geometry, CuKα radiation, energy dispersed detector, and step scan mode). The patterns were registered in the 2θ angle range from 5° to 85° with a step of 0.05°. The accumulation time at each point was 5 s. The estimation of the average crystallite sizes was performed using the Scherrer equation.

### 2.3. Catalytic Cemical Vapor Deposition of 1,2-Dichloroethane

The oxide Ni-Mo-Mg-O sample (10 mg) was loaded into a basket made of foamed quartz. The basket was fixed in a quartz flow reactor equipped with McBain balances as described elsewhere [34,37]. Then, the sample was heated up to 600 °C in a mixed flow of 40 vol% hydrogen in argon (flowrate of 250 mL/min; heating rate of 20 °C/min) and kept at this temperature for 10 min to reduce the metals (Ni and Mo). After the reductive pretreatment procedure, the reaction gas mixture containing 7 vol% 1,2-dichloroethane, 37 vol% hydrogen, and argon (balance) was purged through the reactor. The total flowrate of the reaction mixture was 250 mL/min. The CCVD process was performed for 2 h. When the reactor was cooled down to room temperature, the nanostructure carbon product was unloaded and studied by electron microscopy.

## 3. Results

### 3.1. Characterization of the Prepared Samples

**Figure 1:**
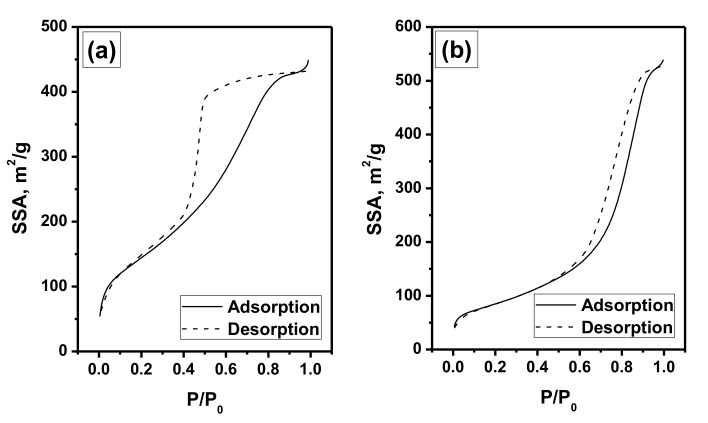
Low-temperature nitrogen adsorption/desorption isotherms of the Ni-Mo-Mg-OH (**a**) and Ni-Mo-Mg-O (**b**) samples.

As known, the developed porous structure of the support (or matrix) determines the dispersion of the supported (distributed) active components and their accessibility for the gas-phase reagents. In this connection, both the xerogel Ni-Mo-Mg-OH and oxide Ni-Mo-Mg-O samples were examined by the low-temperature nitrogen adsorption method. The corresponding isotherms are presented in Figure 1. Type IV isotherms have a hysteresis loop and are typical for the mesoporous adsorbents with capillary condensation in micro- and meso- pores.

The calculated values of the textural parameters are summarized in Table 1. As seen, the prepared samples possessed a porous structure with a developed specific surface area (SSA) and pore volume (V_p_). The values of the average pore size (D_av_) also testified towards the mesoporous structure of the samples. It is quite interesting to compare the obtained values with previously reported ones (Table 1). In the case of pure Mg(OH)_2_ xerogel (denoted as Mg-OH), the SSA value was noticeably bigger. The introduction of nickel or molybdenum into the gel decreased this value to the level of 465–475 m^2^/g. At the same time, these metals being introduced separately showed opposite effects on the pore volume and average pore size. Thus, for the Ni-Mg-OH system, the D_av_ value grew from 40 to 79 Å, while pore volume decreased from 1.25 to 0.92 cm^3^/g. On the contrary, for the Mo-Mg-OH xerogel, pore volume increased to 1.36 cm^3^/g, and D_av_ was significantly larger (114 Å). The calcination of Mg-OH xerogel led to the formation of magnesium oxide with 2.8 times lower SSA, 1.3 times lower pore volume, and ~1.5 times bigger pore size. In the case of two-component oxide systems, the SSA value was 1.6 times lower for Ni-Mg-O, with respect to the Mg-O sample, and 1.4 times higher for Mo-Mg-O. This means that these two metals showed opposite trends affecting the textural parameters of the sol-gel derived M-Mg-O oxides. Being added together, they allowed one to obtain better SSA value than in the case of a pure Mg-O system. Thus, SSA of the Ni-Mo-Mg-O sample was 300 m^2^/g, which is in the middle between values of 154 and 342 m^2^/g, corresponding to the Ni-Mg-O and Mo-Mg-O samples.

**Figure 2:**
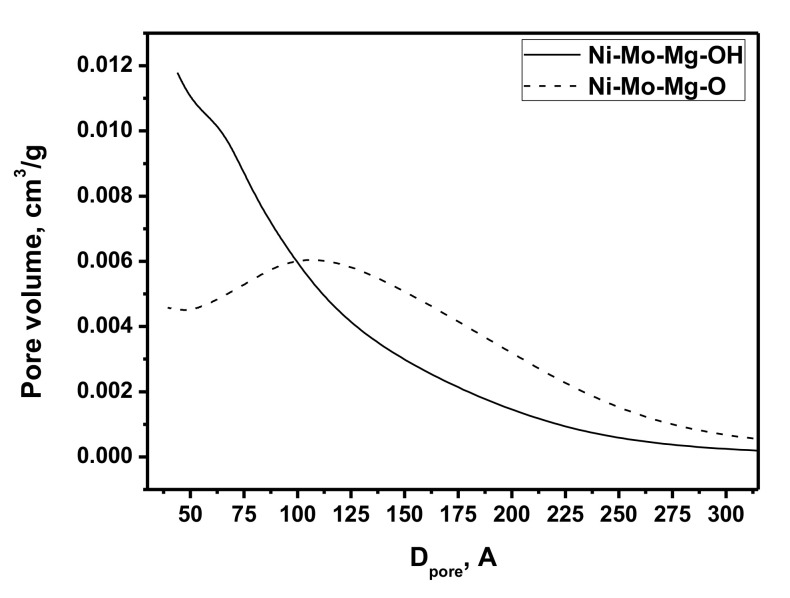
Pore size distributions (Barrett-Joyner-Halenda (BJH) method) of the prepared samples.

Figure 2 demonstrates the pore size distributions obtained from the adsorption curves by the BJH method for the studied samples. The xerogel sample was characterized by not too wide distribution. Small mesopores (up to 10 nm) made the dominant contribution. After the calcination, the portion of larger mesopores increased, and the distribution became wider. Such behavior is explained by the formation of the porous structure of the oxide system. The release of the gaseous products of the Ni and Mo salt precursors decomposition also contributed to the pore-forming process.

**Figure 3:**
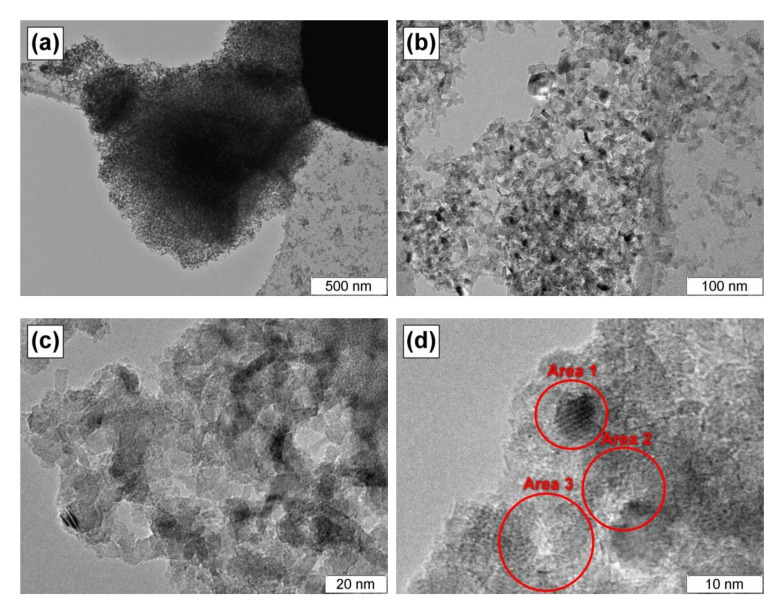
TEM images of the sol-gel prepared Ni-Mo-Mg-O sample at various magnifications: (**a**) ×10,000; (**b**) ×50,000; (**c**) ×250,000 and (**d**) ×500,000.

The Ni-Mo-Mg-O sample was studied by transmission electron microscopy. Figure 3 presents the corresponding TEM images. As seen, the sample is represented by a disordered net consisting of plates of few nm in size. According to the elemental analysis data (Table 2), the darker areas in the images are enriched with nickel and molybdenum compared with other parts. It should also be noted that the small dark dots are quite uniformly distributed within the MgO matrix. As a rule, their appearance testifies for the presence of Mo atoms in the form of clusters. The size of these species is estimated to be from 5 to 10 nm.

**Figure 4:**
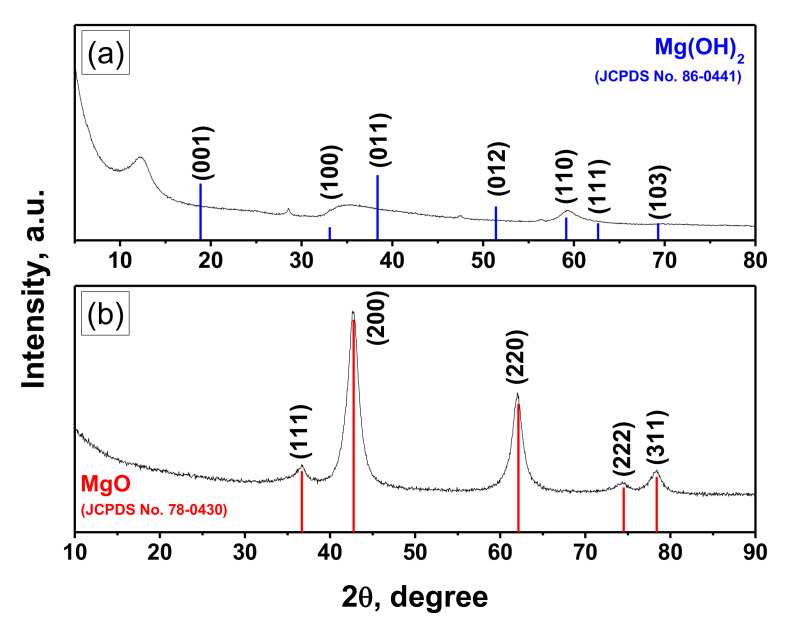
XRD pattern of the sol-gel prepared Ni-Mo-Mg-OH (**a**) and Ni-Mo-Mg-O (**b**) samples.

The XRD pattern of the Ni-Mo-Mg-O sample is shown in Figure 4b. It is evident that all the reflections, by the shape and position, correspond to magnesium oxide. The average MgO crystalline size calculated using the Scherrer equation was found to be of 4 nm, which agrees well with the TEM results. It should be noted that the reflections are slightly shifted towards larger angles, which indicates an increase of the MgO lattice parameters compared with the crystal phase—periclase. One of the possible reasons for this phenomenon is the formation of the solid solution of substitution Ni_x_Mg_1-x_O. Both the oxides belong to the NaCl structural type and have very close lattice parameters: NiO—Fm3m, a = 4.177 Å; MgO—Fm3m, a = 4.211 Å. Therefore, these oxides can easily form solid solutions [32]. No molybdenum-containing phase was observed that presumably testifies towards roentgen-amorphous molybdenum clusters uniformly distributed within the bulk of the oxide matrix.

For comparison, Figure 4a illustrates the XRD pattern of the xerogel Ni-Mo-Mg-OH sample. As seen, the experimentally observed positions of the reflections do not coincide with the reference positions for Mg(OH)_2_. As was recently reported [38], such phenomenon is connected with the unique disordered turbostratic structure of the sol-gel derived magnesium hydroxides.

### 3.2. Catalytic Chemical Vapor Deposition of 1,2–Dichloroethane

**Figure 5:**
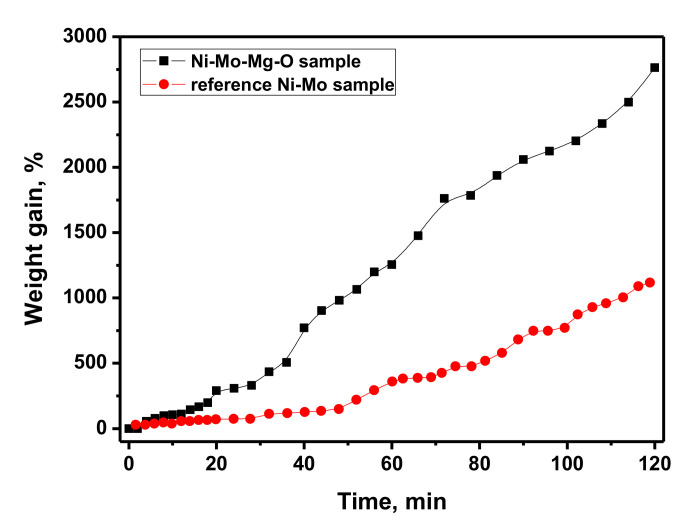
Kinetic curves for the carbon nanofibers formation during the CCVD process of 1,2-dichloroethane.

The catalytic chemical vapor deposition of 1,2-dichloroethane resulting in the formation of carbon nanofibers was performed in a flow-through reactor equipped with McBain balances that allowed the measuring of the weight changes of the catalyst’s sample in a real-time mode. The duration of the experiment was 2 h. The kinetic curve for carbon product deposition is plotted in Figure 5. The weight gain was calculated regarding the mass of metallic Ni and Mo in the sample loaded into the reactor. Another Ni-Mo sample was prepared via the co-precipitation of [Ni(NH_3_)_6_]Cl_2_ and (NH_4_)_6_Mo_7_O_24_ precursors followed by subsequent calcination of the obtained sediment in the reducing atmosphere (H_2_) and served as a reference sample. This sample was represented by a porous Ni-Mo alloy, as reported elsewhere [21]. As can be concluded from the comparison of the kinetic curves (Figure 5), the catalytic performance of the reference sample was characterized by the presence of the so-called induction period (first 20 min), when weight changes are insignificant. During the induction period, the restructuring of the Ni-Mo alloy’s surface and the self-organization of the catalytically active metallic particles take place. Then, after 20 min, the formed particles begin to act as sites of the fibrous carbon growth. In the case of the sol-gel prepared Ni-Mo-Mg-O sample, this activation stage was practically absent. The particles were already formed during the synthesis and subsequent reductive pretreatment. Therefore, the CCVD process occurred, starting from the first minutes. After 2 h of the experiment, the carbon yield over the sol-gel sample exceeded that of the reference sample more than 2.5 times. It can be concluded that the sol-gel technique provided the synthesis of a more efficient catalytic system, which yielded a higher relative amount of nanostructured carbon.

Efficiency of the Ni-based catalysts in relation to various processes is widely discussed in the literature [39]. The porous structure of the support plays a tremendous role in both the activity and stability of the catalysts. On the one hand, the pores should be big enough to provide the accessibility of Ni active sites for the reagents. On the other hand, excessively thin walls of the pores cannot efficiently prevent the high-temperature sintering of nickel particles. In our case, magnesium oxide prepared via the sol-gel route can be considered not as a support but as a matrix represented by an assembly of nanostructured crystallites. The applied approach allowed us to obtain the space-divided particles of the active Ni-Mo phase, thus preventing them from the agglomeration at elevated temperatures. The developed porous structure of the MgO matrix, in its turn, provided good enough accessibility of these particles for 1,2-dichloroethaneand hydrogen molecules.

It should be noted that the conversion of 1,2-dichloroethane was not so high if compared with the traditional catalytic processes. The average value for 2 h of reaction, as estimated from the carbon yield, was found to be about 12.4%. At the same, the conversion value calculated for the last two points in the experimental curve was equal to 23.4%. The reason for such low conversion of the substrate was that the reaction was kinetically limited. Moreover, the mechanism of the 1,2-dichloroethane decomposition over Ni catalysts, as was reported earlier [40], was complicated by the presence of chlorine in the composition of the substrate and, therefore, in the reaction volume. Chlorine species cover the surface of the active particles and temporally poison them, thus excluding them from the participation in the catalytic process. Thereby, an excess of hydrogen is required to clean the catalyst’s surface via the formation of the HCl gaseous product.

**Figure 6:**
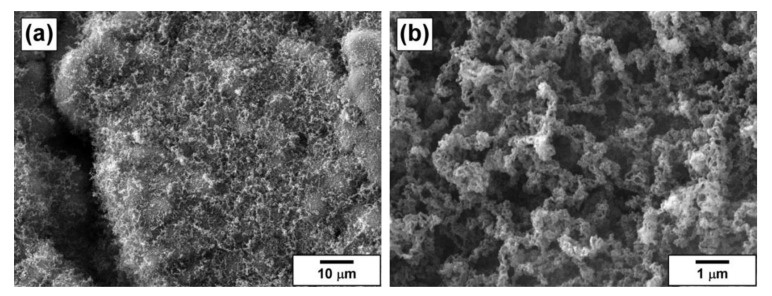
SEM images of the carbon product obtained via CCVD of 1,2-dichloroethane over the Ni-Mo-Mg-O catalyst at various magnifications: (**a**) ×1,000 and (**b**) ×10,000.

The collected carbon product was characterized by low-temperature nitrogen adsorption, and TEM and SEM methods. The SSA value of the carbon nanofibers was found to be 290 m^2^/g, which is typical for the carbon materials of such kind [21]. The results of scanning electron microscopy for the obtained carbon product are presented in Figure 6. The fibrous structure of the product is well seen (Figure 6b). On the whole, the carbon sample reminded a tangle of carbon nanofibers (Figure 6a). The thickness of the fibers was tens nm, which agrees well with the size of Ni and Mo oxide nanoparticles (Figure 3). The fibers possess a corrugated secondary structure. It should be emphasized that the obtained carbon product was uniform in structure and thickness. In the case of conventionally prepared catalysts, the carbon products are usually represented by a set of fibers different in size, structure, and morphology. Therefore, one more advantage of the sol-gel approach is the possibility to control the uniformity of the synthesizing carbon materials.

**Figure 7:**
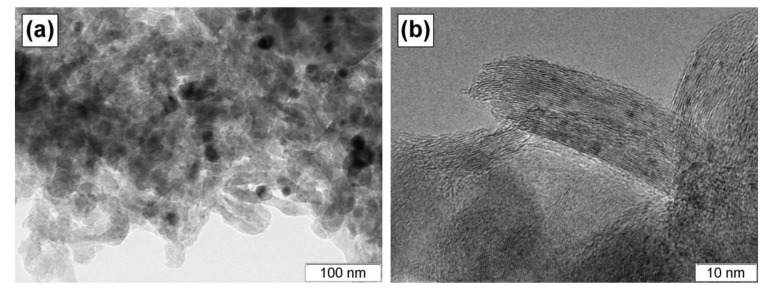
TEM images of the carbon product obtained via CCVD of 1,2–dichloroethane over the Ni-Mo-Mg-O catalyst at various magnifications: (**a**) ×50,000 and (**b**) ×500,000.

In terms of morphology, the carbon fibers belong to a feathery-like type. Such type is characterized by a disordered structure when graphene packages being chaotically oriented form the fiber. The TEM image of the carbon nanofibers is shown in Figure 7a. The black dots in this image are Ni-Mo catalytic particles. The fibers are comprised of the graphene packages illustrated in Figure 7b. Each package consists of 20 to 40 layers and has a size of ~10 nm in width and ~50 nm in length. The attractiveness of these carbon nanofibers is connected with their softness and easy collapsibility. Due to the mentioned unique properties, these materials are very promising for application in lubricants as fillers.

## 4. Conclusions

The three-component Ni-Mo-Mg-O system was prepared via a sol-gel approach and characterized by a set of physicochemical methods. It was shown that the obtained samples possessed the developed structure and high values of specific surface area and pore volume. From a morphological point of view, the samples are composed of plates of a few nm in diameter. According to the elemental analysis results, the dark areas evenly distributed within the MgO matrix are enriched with Ni and Mo atoms. The XRD study of the samples revealed that the reflections corresponding to magnesium oxide were shifted towards larger angles. This testified for the formation of the solid solution Ni_x_Mg_1-x_O. No additional phases were observed that allow us to conclude that the active components were uniformly distributed within the oxide matrix in a roentgen-amorphous state. The sol-gel prepared Ni-Mo-Mg-O sample was compared with the conventionally prepared reference one in the catalytic chemical vapor deposition of 1,2-dichloroethane. The sol-gel catalyst was found to be more efficient in this process, producing the carbon product with a higher yield. The deposited carbon was represented by nanosized fibers of even diameter, thus confirming the uniformity of the metal distributions in the matrix. Each carbon fiber was composed of chaotically oriented graphene packages, which, in their turn, consisted of 20–40 layers.

## Figures and Tables

**Table 1:** Textural properties of the prepared and previously reported samples.

Sample	SSA, m^2^/g	V_p_, cm^3^/g	D_av_, Å	Ref.
Ni-Mo-Mg-OH	520	0.77	53	This work
Ni-Mo-Mg-O	300	0.85	111	This work
Mg-OH	680	1.25	40	[31]
Mg-O	243	0.97	61	[31]
Ni-Mg-OH	465	0.92	79	[31]
Ni-Mg-O	154	0.72	189	[31]
Mo-Mg-OH	475	1.36	114	[31]
Mo-Mg-O	342	1.30	153	[31]

**Table 2:** EDX data for the areas shown in Figure 3d.

Area	Content, %		
	Mg	Ni	Mo
1	67.5	20.7	11.8
2	71.5	19.4	9.1
3	88.6	4.6	6.8
